# Does Hepcidin Tuning Have a Role among Emerging Treatments for Thalassemia?

**DOI:** 10.3390/jcm11175119

**Published:** 2022-08-30

**Authors:** Filomena Longo, Antonio Piga

**Affiliations:** 1 Thalassemia Reference Centre, 10043 Orbassano, Italy; 2 Regional HUB Centre for Thalassaemia and Haemoglobinopathies, Department of Medicine, Azienda Ospedaliero Universitaria S. Anna, 44124 Ferrara, Italy; 3 University of Torino, 10043 Torino, Italy

**Keywords:** hepcidin, ferroportin, thalassemia, erythropoiesis

## Abstract

The treatments available for thalassemia are rapidly evolving, with major advances made in gene therapy and the modulation of erythropoiesis. The latter includes the therapeutic potential of hepcidin tuning. In thalassemia, hepcidin is significantly depressed, and any rise in hepcidin function has a positive effect on both iron metabolism and erythropoiesis. Synthetic hepcidin and hepcidin mimetics have been developed to the stage of clinical trials. However, they have failed to produce an acceptable efficacy/safety profile. It seems difficult to avoid iron over-restricted erythropoiesis when directly using hepcidin as a drug. Indirect approaches, each one with their advantages and disadvantages, are many and in full development. The ideal approach is to target erythroferrone, the main inhibitor of hepcidin expression, the plasma concentrations of which are greatly increased in iron-loading anemias. Potential means of improving hepcidin function in thalassemia also include acting on TMPRSS6, TfR1, TfR2 or ferroportin, the target of hepcidin. Only having a better understanding of the crosslinks between iron metabolism and erythropoiesis will elucidate the best single option. In the meantime, many potential combinations are currently being explored in preclinical studies. Any long-term clinical study on this approach should include the wide monitoring of functions, as the effects of hepcidin and its modulators are not limited to iron metabolism and erythropoiesis. It is likely that some of the aspects of hepcidin tuning described briefly in this review will play a role in the future treatment of thalassemia.

## 1. Introduction

Thalassemia and Sickle Cell Disease (SCD) are among the most common inherited disorders, affecting millions of people worldwide [1,2].

**Figure 1:**
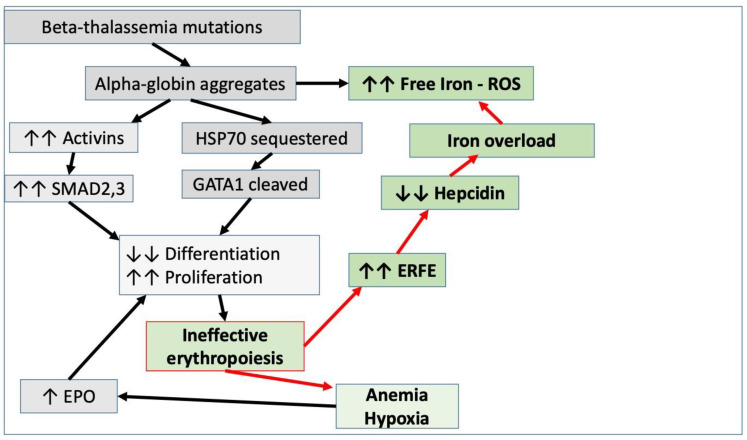
Central role of ineffective erythropoiesis in hepcidin cascade in thalassemia (ERFE = erythroferrone; EPO = erythropoietin; ROS = reactive oxygen species).

β-thalassemia is caused by mutations in the β-globin gene that lead to unbalanced α- and β-chain production, with the expansion of abnormal erythroid precursors, maturation blockage and increased apoptosis of late erythroblasts (Figure 1). This detrimental condition of ineffective erythropoiesis (IE) is tightly linked to iron metabolism dysregulation with hepcidin suppression, which predisposes patients to severe iron loading even in the absence of transfusions [3]. Thalassemia is the prototype of the so-called iron-loading anemias.

The degree of anemia and IE influence different phenotypes. Thalassemia major (TM) is defined as a severe form of anemia with early bone alterations and lifelong transfusion need; thalassemia intermedia (TI) includes a broad spectrum of severity, from very mild conditions to moderate ones with a high risk of clinical complications. Regarding basic treatment, thalassemias are grouped as transfusion-dependent β-thalassemia (TDT) and non-transfusion-dependent β-thalassemia (NTDT) [4]. The TM/TI and TDT/NTDT categories are not synonymous but complementary; experienced clinicians label a patient permanently as TM or TI, according to the clinical presentation and the severity of genetic defects. On the contrary, the TDT/NTDT label may vary according to local blood availability and individual clinical course. A significant proportion of TI patients move sooner or later from NTDT to TDT due to worsening anemia or for preventing/controlling complications. In general, both TDT and most NTDT patients suffer from iron overload, which requires chronic iron chelation therapy to prevent iron-related complications, such as endocrinopathies and liver and heart disease [4].

Sickle Cell Disease (SCD) is a monogenic disorder that results in progressive multiorgan disease due to intravascular hemolysis, acute vaso-occlusive crisis and chronic inflammation. If, in SCD, on the one hand, anemia leads to the suppression of hepcidin, on the other hand, inflammation and chronic hemolysis upregulate hepcidin. The final result of these two patterns in each patient may vary widely, but a minority of SCD patients have a pattern of iron-loading anemias [5].

## 2. Hepcidin–Ferroportin Axis

Hepcidin is a peptide hormone produced by the HAMP gene (19q13), initially associated with in vitro antimicrobial activity only [6,7]. Then, hepcidin emerged as the key hormone for iron homeostasis regulation [8]. Encoded as a pro-peptide by the cleavage of two isoforms, hepcidin-25 represents the active one, with an N-terminal residue of five amino acids that are crucial for iron regulation, inhibition of dietary iron absorption and iron release from storage [9]. Hepcidin is primarily synthesized in the hepatocytes and, to a lesser extent, by monocytes [10], macrophages [11], adipocytes [12], the kidneys [13], the small intestine, the placenta [14], retinal cells [15] and cardiomyocytes [16]. Hepcidin is upregulated by iron levels [17], inflammation and infection [18,19], while anemia, hypoxia, iron deficiency, ineffective erythropoiesis and increased erythropoietin (EPO) levels are down-regulating factors [19,20]. Hepcidin synthesis is mainly controlled throughout the bone morphogenetic proteins BMP6 and, to a lesser extent, BMP2 produced by endothelial cells, which activate signal transmission through SMAD proteins (Figure 1) [21,22,23]. Once secreted into the circulation, hepcidin binds to ferroportin (FPN), the only known cellular iron exporter in vertebrates, localized at the basolateral membranes of duodenal enterocytes [24]. FPN is also abundantly expressed on cells that recycle iron from senescent red blood cells, such as macrophages of the spleen, bone marrow and liver [24]. Hepcidin binding to ferroportin is coupled to iron binding, with an 80-fold increase in hepcidin affinity in the presence of iron [25]. Hepcidin binding occludes the central cavity of FPN, interfering with iron export independently of endocytosis [26]. The iron-related differential affinity for FPN has the important function of determining the inhibition of cells actively involved in iron export, such as intestinal cells and macrophages [25]. After hepcidin binding, FPN is internalized and degraded by lysosomes, requiring the resynthesis of degraded ferroportin molecules. The final result is that the outflow of iron from cells is reduced, and the intracellular storage increases [27]. Hepcidin’s effect lasts up to 48 h.

The massive erythron expansion of thalassemia requires up to 100-fold the physiological daily iron amount [28,29]. This requirement is met by the increased expression of erythroferrone (Erfe), a recently identified iron-regulatory hormone highly expressed in erythroblasts in response to EPO stimulation [30]. Erfe-knockout mice are not anemic, indicating that Erfe has a modest effect on hepcidin repression in steady state. However, Erfe contributes to iron loading in mice with β-thalassemia [31]. Erfe acts as a potent hepcidin suppression, thus enhancing duodenal iron absorption and iron mobilization from stores. This favors iron availability for the increased erythropoietic demand. The erythroid sensing, throughout Erfe, prevails over the iron sensing. This aspect is useful in physiological conditions, but, in thalassemias, it is responsible for triggering iron overload, both in NTDT and TDT patients [20,31,32,33]. 

The most potent Erfe inhibitor is the liver transmembrane serine protease TMPRSS6 (transmembrane serine protease 6) or matriptase-2 TMPRSS6, which is mutated in patients with iron-refractory iron deficiency anemia (IRIDA) [34]. TMPRSS6 cleaves the BMP coreceptor hemojuvelin (HJV), thereby avoiding the overactivation of BMP/SMAD signaling and hepcidin transcription [35].

The expansion of the erythropoietic compartment is backed by the expression of transferrin receptor 1 (TfR1) and transferrin receptor 2 (TfR2), which modulate the iron supply to the erythron [36]. TfR1 is the most abundant cell membrane protein on erythroblasts and is essential in mediating transferrin endocytosis. 

Unfortunately, a simple and direct marker of erythropoiesis is lacking. This makes intra- and interpatient comparisons, with or without treatment, challenging in thalassemia. Recently, the ratio of sTfR1 plasma concentration (proportional to the volume of erythroid tissue) divided by absolute reticulocyte count (proportional to effective erythroid output from the bone marrow) has been proposed as the best index to quantify ineffective erythropoiesis [37]. In mouse models of beta-thalassemia, decreased expression of TfR1 has been associated with improved iron metabolism and IE [38]. TfR2, a constitutive component of the Epo receptor complex, is expressed during the differentiation of erythroid cells [39], but its function has not been fully unveiled. Even so, TfR2 inhibition has potential therapeutic implications as, in thalassemic mice, the knockout of TfR2 lowers Epo and Erfe levels, increasing the proportion of mature precursors and hemoglobin [40]. 

Thalassemia is the prototype of iron-loading anemias. Urinary hepcidin levels are suppressed in patients with NTDT not receiving transfusions. Hepcidin is also low, but to a less pronounced extent, in patients on a regular transfusion program, even with more severe iron overload [41]. This finding is not surprising, as transfusion improves anemia and suppresses erythropoiesis, with an increase in hepcidin production [42]. However, hepcidin concentrations progressively decrease in the intervals between transfusions [43,44]. In summary, any severe form of thalassemia, even on regular transfusion, should be considered an iron-loading anemia.

## 3. Targeting the Hepcidin–Ferroportin Axis in Thalassemia

Restoration of hepcidin levels in thalassemia could reduce iron absorption, improve ineffective erythropoiesis and possibly, in the long term, also reduce iron toxicity [45]. Proof-of-principle studies indicate that a moderate increase in hepcidin expression reduces tissue iron levels, with a beneficial effect on erythropoiesis, in an NTDT mouse model [46].

### 3.1. Hepcidin Mimetics

Two hepcidin mimetics (LJPC-401 and PTG-300, see Table 1) have been developed independently and have demonstrated efficacy in preclinical models. Phase 1 studies on humans showed a significant and long-lasting reduction in serum iron following their administration [47]. LJPC-401 is a synthetic, full-length hepcidin mimetic with the mature form of the human hepcidin. It reached a phase 2 study, primarily aiming at evaluating its efficacy in cardiac iron reduction in adult TDT patients with myocardial iron overload (NCT03381833). The study failed to produce an acceptable efficacy/safety profile and further development was foregone. A slightly different approach was applied to synthesize PTG-300, a hepcidin mimetic that shares only the N-terminal portion of the human peptide sequence [48]. PTG-300 was investigated in a phase 2 clinical trial on TDT and NTDT patients (NCT03802201) that confirmed its ability in reducing transferrin saturation and serum iron [49]. Unfortunately, for PTG-300, injection site reactions have been a significant limitation. The so-called mini-hepcidins are truncated hepcidin peptides based on the *N*-terminal sequence of hepcidin and have several potential advantages, but, at present, none has entered clinical development. However, preclinical results in an NTDT mouse model are promising [50]. In addition, in a new mouse model with a severe transfusion-dependent thalassemia disease phenotype, a mini-hepcidin (MH), combined with red blood cell transfusion, ameliorated IE, splenomegaly and cardiac iron overload [51]. Recently, new mini-hepcidins with improved drug-like properties have been designed using head-to-tail cyclization and *N*-methylation [52].

### 3.2. Hepcidin Agonists

Another approach to the search for hepcidin agonist action is indirect, by inhibiting the main regulators of hepcidin function, such as TMPRSS6 and Erfe, or by inhibiting the hepcidin target ferroportin [23].

- *TMPRSS6 inhibition*

In an NTDT mouse model, loss of TMPRSS6 significantly attenuated the disease phenotype [53]. Two independent studies, using oligonucleotides such as antisense oligonucleotides (ASO) or small interfering RNA (siRNA), have confirmed these findings, not only decreasing iron loading but also improving erythropoiesis, splenomegaly and anemia [54,55]. Two phase 2 clinical trials on NTDT patients are currently ongoing, using IONIS TMPRSS6-LRx (NCT04059406) and SLN124 (NCT04718844). Recently, the anti-TMPRSS6 antibody MWTx-003 was presented as a promising therapy for iron overload disorders where an iron restriction is beneficial (Bruxin, ASH 2021).

- *Ferroportin inhibition*

Advances in understanding the structure of hepcidin-bound ferroportin and its iron homeostatic mechanisms [25] opened the door to a new approach: targeting ferroportin directly. This offers great potential in tuning the restriction of cellular iron export. Many compounds have been screened for this function. The first to enter development has been vamifeport (VIT-2763), a small molecule orally administered, that acts as an inhibitor of ferroportin, competing with hepcidin for binding to ferroportin [56]. In healthy volunteers, a temporary decrease in mean serum iron levels, transferrin saturation and a shift in mean serum hepcidin peaks followed the administration of VIT-2763 [57]. A phase 2 study of VIT-2763 in NTDT (NCT04364269) is ongoing.

- *Erfe inhibition*

In theory, Erfe inhibition is the best approach to tune hepcidin function for iron-loading anemias, because the function of Erfe is to tune hepcidin merely based on erythroid needs. In a thalassemia mouse model, Erfe inhibition blocked its suppressive effects on hepcidin, with amelioration of the phenotype [31]. Furthermore, due to its distribution and picomolar concentration, Erfe is the ideal target for monoclonal antibodies. This approach, using *N*-terminal neutralizing antibodies, has been recently applied with promising results in thalassemia mice [58].

- *TfR2 inactivation*

The inactivation of the EPO receptor partner, TfR2, in a knockout thalassemia intermedia mouse model improved erythropoiesis and red blood cell morphology, as well as anemia and iron overload [40]. However, the beneficial effects became attenuated over time, possibly due to insufficient iron availability to sustain the enhanced erythropoiesis. Germline deletion of TfR2, including haploinsufficiency, has a similar impact in the thalassemic model [40]. Data from TfR2-haploinsufficient thalassemic mice suggest that TfR2-specific targeting by antisense oligonucleotides or small interfering RNAs has great therapeutic potential in NTDT [59]. A better understanding of the TfR2–EPOR interaction may lead to the design of interfering molecules, mimicking an erythroid-specific TfR2 depletion. Unlike erythropoiesis-stimulating agents, the TfR2 approach enhances EPO-mediated effects exclusively in erythroid cells, with potential advantages for long-term safety [59].

- *Apotransferrin*

In thalassemic erythropoiesis, iron is abundant due to the massive erythroid demand and hepcidin suppression, but is underutilized due to the genetic defect of hemoglobinization. As the iron turnover is high, the therapeutic use of apotransferrin has been hypothesized. In NTDT mice, apotransferrin infusion improved anemia, splenomegaly and plasma EPO, decreased membrane-associated α- globin precipitates and normalized the RBC half-life [38]. In addition, a reduction in cardiosiderosis has also been observed [60]. A phase 2 clinical trial using i.v. infusion of apotransferrin every other week is currently being performed in beta-thalassemia (NCT03993613).

### 3.3. Combination Therapy

The availability of several therapeutic tools targeting different pathways has allowed the exploration of balanced combinations, searching for a potential additive or even synergistic effect (Table 2). For example, in NTDT mice, TMPRSS6 inhibition (by TMPRSS6-ASO or siRNA) combined with the iron-chelating agent deferiprone produced some additive effects on the improvement of erythropoiesis and iron overload [61,62,63]. Another interesting approach has been assessed by combining TMPRSS6 inhibition with EPO administration or removing a single TfR2 allele in the bone marrow of NTDT mice. Both combinations were more effective than a single agent in ameliorating anemia and splenomegaly [59]. Moreover, the inhibition of both TMPRSS6 and TfR2 gave interesting results in Hbbth3/+ thalassemic mice: even with the iron overload due to the TfR2 double mutation, a therapeutic effect on both erythropoiesis and anemia was obtained [64]. Another recent approach combined a direct ferroportin inhibitor (VIT-2763) with an iron chelator (deferasirox) [65]. The promising results in NTDT herald potential clinical trials. Of many other possible combinations, several are worthwhile at least in vitro testing, given the potentially strong rationale, such as Erfe inhibition with iron chelation.

## 4. The Background of Treatment Options

### 4.1. Conventional Treatment

Severe thalassemia requires regular transfusions and daily iron chelation treatment, accompanied by high-quality monitoring and follow-up. The latter is often enough in TI to preserve good quality of life and prevent long-term thalassemia-related complications such as bone alterations, hypersplenism, thrombotic events, leg ulcers and acquired elastopathy. Where quality of care and patient adherence is kept optimal, the long-term results in terms of survival and quality of life are good, with a life expectancy that approaches normality. However, many literature reports indicate the significant prevalence of long-term complications due to poor adherence and consequent poor control of iron overload-related toxicity.

### 4.2. Stem Cell Transplantation

A standard approach to the conditioning regimen and immunosuppression has been set for time in different risk classes [66,67]. The likelihood of success in the low-risk category is more than 90% and, differently from the past, it is not limited to pediatric subjects. Therefore, balanced counselling is important to weigh the advantages and disadvantages of conventional treatment versus stem cell transplantation in each individual, especially in the intermediate risk class and according to local health resources. 

### 4.3. Gene Therapy

The gene therapy approach in hemoglobinopathies was introduced many years ago [68], but only in recent times has it seen an impressive acceleration in terms of clinical trials, up to the first approval by regulatory agencies [69]. Today, the classical gene addition is performed by lentiviral vector insertion into stem cells after a myeloablative conditioning regimen [70,71]. Impressive results regarding the efficacy [71] are counterbalanced by long-term safety issues in the light of recent complications with cases of leukemia and myelodysplasia in an SCD trial [72]. The impact of disease-related rather than procedure-related complications is still unclear. Recently, the hypothesis has emerged that the stress hematopoiesis in transplanted cells may drive clonal expansion and the leukemogenic transformation of preexisting premalignant clones [73]. At present, the authors of this review believe that, with the exclusion of ongoing trials, the gene addition approach for hemoglobinopathies will not experience significant development. 

On the contrary, the genome editing approach is developing rapidly, with great expectations in many fields of medicine, including hemoglobinopathies. At present, for both thalassemia and Sickle Cell Disease, rather than repairing the causative mutations, the simpler approach of knocking out the lineage-specific regulatory element of the BCL11A gene is under intense investigation. Suppressing BCL11A potently affects fetal hemoglobin reactivation, mitigating or cancelling the pathological impact on both disorders. Among several techniques, CRISPR-Cas9-based genome engineering is by far the most applied one. Several clinical trials are ongoing in thalassemia (NCT03432364, NCT03655678, NCT04208529) and SCD (NCT03745287, NCT04819841), with impressive preliminary results as regards the efficacy. In terms of safety, even if several issues have been solved, concerns associated with the nature of this technique (double strains cut and predisposition to deletions) are still unresolved. It is likely that the full approval of the genome editing approach for treating hemoglobinopathies will require several years of intensive research. 

In general, in the case of potential access to gene therapy, the patient needs balanced counselling that takes into account first the access to and quality of conventional treatment. When full access and high quality are available, prudence is due. For a patient living in a country with inadequate care, the likelihood of stem cell transplantation or a gene therapy approach is balanced by the risk of poor conventional treatment. Obviously, these considerations will remain theoretical if the high cost of gene therapy does not lower consistently. 

### 4.4. Modulation of Erythropoiesis and Erythrocyte Metabolism

**Figure 2:**
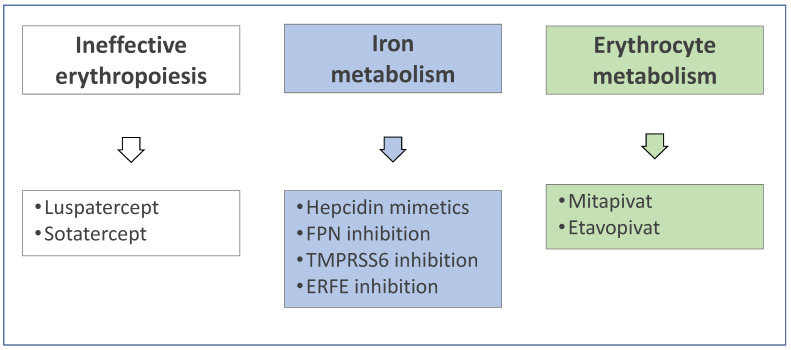
Potential new treatments emerging from advances in the pathophysiology of thalassemia (ERFE = erythroferrone; FPN = ferroportin; TMPRSS6 = transmembrane serine protease 6).

Given the present limitations of conventional treatment, stem cell transplantation and gene therapy described above, any intervention able to significantly ameliorate the erythropoiesis in hemoglobinopathies is meaningful. Several potential new treatments are emerging from advances in the pathophysiology of thalassemia targeting ineffective erythropoiesis, iron or erythrocyte metabolism (Figure 2).

- Mitapivat is an oral, small-molecule allosteric activator of pyruvate kinase in red blood cells [74] that has been very recently approved as a treatment for hemolytic anemia in adults with pyruvate kinase (PK) deficiency. PK is functionally normal but low in thalassemic red blood cells, and mitapivat, in a mouse model of thalassemia, demonstrated an interesting improvement in ineffective erythropoiesis and anemia [75]. A phase II study in beta and alpha thalassemia patients showed encouraging results [76]. A clinical trial is ongoing in TDT (NCT04770779). Etavopivat is an analogue compound in development to treat SCD and thalassemia [77].
- Luspatercept has been recently approved for TDT patients in Europe, the United States and other countries [78,79]. It acts as a trap of the soluble ligands of activin receptor type 2, eventually resulting in more effective RBC differentiation (Figure 1). The clinical result is a consistent reduction in the transfusion burden. Preliminary results from ongoing trials in NTDT indicate a consistent increase in total hemoglobin (NCT04064060).

## 5. Discussion

As hepcidin has a paramount role in iron homeostasis, its targeting has great potential, especially in conditions where hepcidin is deeply suppressed, as in iron-loading anemias, including thalassemias. As a small peptide, it appears natural to include hepcidin in the long list of synthetic peptide hormones successfully developed and used today as effective drugs in the clinical setting. At present, however, this does not appear likely for hepcidin or hepcidin mimetics, due to challenges in achieving a stable compound, the need for frequent subcutaneous injections and the frequency of site reactions. More importantly, it is difficult to tune their effect, avoiding iron over-restricted erythropoiesis. When the therapeutic window is narrow, the general rule is to turn to indirect tuning. This has been the case for hepcidin agonists, with many studies and interesting results presented regarding single agents and combinations. The first to be used in clinical trials was TMPRSS6, and many other new entries can be expected. As the effects of hepcidin and its modulators are not limited to iron metabolism and erythropoiesis, it is important to develop long-term studies examining potential positive and negative consequences. For example, the trend towards osteoporosis in thalassemia is well known and important. Using hepcidin or Erfe in animals, there have been some negative findings on bone markers that, if confirmed, pose a challenge for their long-term use. It is difficult today to define the precise position of hepcidin tuning in the rapidly expanding pipeline of treatment options for thalassemias. Excluding a curative effect, we can forecast a relevant role in lowering the burden of the disease and improving the phenotype, as is happening for luspatercept, alone and in combination.

## 6. Conclusions

Hepcidin tuning reached the phase of clinical development thanks to the progress of our understanding on the crosslinks between iron metabolism and erythropoiesis. Advanced clinical trials on different aspects of hepcidin tuning are currently underway. It is likely that this type of approach will play a role in the future treatment of thalassemia. 

## Figures and Tables

**Table 1:** Treatments to increase hepcidin function in thalassemia (TD = transfusion-dependent; NTD = non-transfusion-dependent; ERFE = erythroferrone; FPN = ferroportin; HSCT = hematopoietic stem cell transplantation; TMPRSS6 = transmembrane serine protease 6; ASO = antisense oligonucleotide; siRNA = small interfering RNA; NTBI = non-transferrin-bound iron; TfR2 = transferin receptor 2; TfR1 = transferin receptor 1; BM = bone marrow).

Action	Mechanism		Compound/Model	Thal Type	CTs?	Clinicaltrials.gov *or Key Reference*
Hepcidin mimetic	Hepcidin mimetics		-Hepcidin LJPC-401 -Hepcidin PTG-300 -Hepcidin PTG-300 -Mini-hepcidin	TD TD TD/NTDT	YES YES YES NO	-NCT03381833 -NCT03802201 -NCT04054921 *-Goncalves, 2021*
Hepcidin agonist	TMPRSS6 inhibition	-by RNA ASO -by siRNA	-Ionis-TMPRSS6-LRx -SLN124	NTD NTD	YES YES	-NCT04059406 -NCT04718844
	FPN inhibition		VIT-2763	NTD	YES	-NCT04364269
	ERFE inhibition		N-Terminal ERFE Abs		NO	*Arezes, 2020*
	TfR2 inactivation		BM TfR2KO mice		NO	*Artuso, 2018*
Apotransferrin	↓ TfR1 expression ↓ NTBI		Human apotransferrin	NTD	YES	-NCT03993613

**Table 2:** Potential combination of treatments targeting hepcidin function (TD = transfusion-dependent; NTD = non-transfusion-dependent; ERFE = erythroferrone; FPN = ferroportin; HSCT = hematopoietic stem cell transplantation; TMPRSS6 = transmembrane serine protease 6; ASO = antisense oligonucleotide; siRNA = small interfering RNA).

Combination				
1	2	Condition	Findings	Ref.
TMPRSS6 inhibition	Iron chelation (Deferiprone)	Hb^bth3/+^ mice	↓ Liver iron	Vadolas J, 2021
TMPRSS6 inhibition	TfR2 inhibition (Tfr2^Y245X/Y245X^double mutant)	Hb^bth3/+^ mice	↑ Hb ↓ Tissue iron	Schmidt PJ, 2020
TMPRSS6 inhibition	EPO	Hb^bth3/+^ mice	↑ Hb ↓ splenomegaly	Casu C, 2020
TMPRSS6 inhibition	TfR2 single-allele deletion	Hb^bth3/+^ mice	↑ Hb ↓ splenomegaly	Casu C, 2020
FPN inhibition	Iron chelation (Deferasirox)	Hb^bth3/+^ mice	↑ Hb ↓ Liver iron	Nyffenegger N, 2021
